# Clinical significance of human metapneumovirus detection in critically ill adults with lower respiratory tract infections

**DOI:** 10.1186/s13613-023-01117-w

**Published:** 2023-03-20

**Authors:** Natacha Kapandji, Michael Darmon, Sandrine Valade, Maud Salmona, Jérôme Legoff, Lara Zafrani, Elie Azoulay, Virginie Lemiale

**Affiliations:** 1 Medical ICU, Saint Louis Academic Hospital, APHP, 1 Avenue Claude Vellefaux, 75010 Paris, France; 2 Virology department, Saint Louis Hospital, Paris, France

**Keywords:** Human Metapneumovirus, Pneumonia, Coinfection, Immunocompromised

## Abstract

**Background:**

Unlike other viruses, the pathogenicity of human metapneumovirus (hMPV) in adults remains uncertain. To address this question, a retrospective monocentric cohort including all patients admitted to ICU with hMPV infection between January 1, 2010, and June 30, 2018 was performed. The characteristics of hMPV infected patients were studied and compared to matched influenza infected patients. Consecutively, a systematic review and meta-analyses investigating PUBMED, EMBASE and COCHRANE databases was conducted to explore the hMPV infections in adult patients (PROSPERO number: CRD42018106617). Trials, case series, and cohorts published between January 1, 2008 and August 31, 2019 compiling adults presenting hMPV infections were included. Pediatric studies were excluded. Data were extracted from published reports. Primary endpoint was the rate of low respiratory tract infections (LRTIs) among all hMPV infected patients.

**Results:**

During the study period, 402 patients were tested positive for hMPV. Among them 26 (6.5%) patients were admitted to the ICU, 19 (4.7%) for acute respiratory failure. Twenty-four (92%) were immunocompromised. Bacterial coinfections were frequent 53.8%. Hospital mortality rate was 30.8%. In the case–control analysis, the clinical and imaging characteristics were not different between hMPV and influenza infected patients. The systematic review identified 156 studies and 69 of them (1849 patients) were eligible for analysis. Although there was heterogeneity between the studies, the rate of hMPV LRTIs was 45% (95% CI 31–60%; *I*^2^ = 98%). Intensive care unit (ICU) admission was required for 33% (95% CI 21–45%; *I*^2^ = 99%). Hospital mortality rate was 10% (95% CI 7–13%; *I*^2^ = 83%) and ICU mortality rate was 23% (95% CI 12–34%; *I*^2^ = 65%). Underlying malignancy was independently associated with increased mortality rate.

**Conclusions:**

This preliminary work suggested that hMPV may be associated with severe infection and high mortality in patients with underlying malignancies. However, regarding the small size of the cohort and the heterogeneity of the review, more cohort studies are warranted.

**Supplementary Information:**

The online version contains supplementary material available at 10.1186/s13613-023-01117-w.

## Background

Viruses are increasingly identified as an etiology for severe respiratory infections. Since 2009, the seasonal flu accounts for high proportions of intensive care unit (ICU) admissions every year and recently the SARS-CoV-2 has led to the worldwide pandemic. Pathogenicity has been well-described for such viruses, but for others such as human metapneumovirus (hMPV) it remains controversial. hMPV was described in 2001 [1]. As human respiratory syncytial virus, hMPV belongs to the pneumoviridae family [2]. The virus is circulating worldwide [3], with a peak incidence in the late winter and beginning of spring slightly delayed from influenza and human respiratory syncytial virus epidemics [4]. hMPV was first detected in children during bronchiolitis and acute asthma exacerbations, but also in severe low respiratory tract infections mainly in immunocompromised children [5]. In adults settings, hMPV was detected in flu-like syndromes [5]. Recently, the increased use of PCR respiratory panels in ICU admitted patients has led to more hMPV detection. However, descriptions of hMPV infection in adult setting remains scarce. We conducted this retrospective cohort study to describe the clinical and radiological characteristics of patients admitted to the ICU with hMPV infection. To explore its pathogenicity, we compare these characteristics to those of patients with influenza infection. Consecutively, a systematic review and meta-analyses was conducted to explore impact of hMPV infection in adult patients: the lower respiratory tract infections (LRTIs), the ICU admission and the mortality rates were explored.

## Materials and methods

### Cohort study and case–control analysis

The study was performed in a single center, particularly involved in onco-hematologic patients. The study was approved by the ethic committee of the Société de Réanimation en Langue Française and informed consent was waived according to French law (CE-SRLF 20-11). hMPV detection was made on upper respiratory tract swabs performed according to the clinicians’ decision. The respiratory panel used was the Respifinder SMART 22 FAST (Eurogentec®) until 2016 then the FilmArray Respiratory v1.7 (Biofire®). All patients tested positive for hMPV between September 1, 2010, and June 30, 2018, were retrospectively screened for inclusion. Those admitted to the ICU were included in the cohort. Control patients were admitted to the ICU during the same time frame and tested positive for influenza A, influenza AH1N1 or influenza B. Matching was performed using the local database considering age (± 5 years), admission year, and underlying hematologic malignancy. hMPV patients presenting viral coinfections and patients admitted to the ICU for other reason than acute respiratory failure were not included in the pairing process. When pairing was not possible, cases were excluded from the matching analysis. All clinical data were retrospectively collected from medical charts: age, sex, comorbidities, medications, clinical, biological, chest imaging characteristics at ICU admission, length of ICU and hospital stay, and mortality at hospital discharge. Regarding clinical symptoms, flu-like syndrome was defined as the association of fever, myalgia, and headache. Coinfection was considered when other pathogens (virus, bacteria, or fungi) were detected on respiratory samples within 1 day before and 3 days after the diagnosis of hMPV infections. First chest X-rays after ICU admission was analyzed as well as the closest chest Computed Tomography (CT) scan to the day of hMPV infection diagnosis. Imaging patterns were classified as alveolar consolidation or interstitial pattern according to the predominant anomaly on chest X ray. Pleural effusion, and the number of quadrants involved were also recorded. CT-scans were described regarding several patterns: alveolar consolidations, ground glass opacities, crazy-paving, septal thickening, and bronchial lesions. Chest X-ray and CT-scans were independently analyzed by two intensivists (NK, VL). In case of a mismatch, interpretation was discussed until agreement. All data were expressed as n (%) or median (IQR) as required. Clinical characteristics of hMPV infected patients were first described. Then, comparison between hMPV and influenza infected patients was performed by univariate analysis. Considering the small sample size, parametric tests were performed, and multivariate analysis could not be conducted. All statistical analyses were performed using the R statistical Software (version 3.3.2, R Foundation for statistical computing, Vienna, Austria).

### Systematic review and meta-analyses

#### Search strategy and selection criteria

PUBMED, EMBASE, and COCHRANE databases were systematically searched between January 1, 2008 and July 31, 2019 using structured search equations (Additional file 1: Appendix 1). Trials, case series, prospective, and retrospective cohorts investigating adult patients with confirmed hMPV infection were included whatever test was used to confirm the hMPV infection. Studies including pediatric patients (under 18 years) were excluded. Meta-analyses, reviews, studies evaluating new diagnosis tests, fundamental research on vaccinology, and epidemiologic reports were not included. The research was restricted to English-written abstracts with full-text articles available. Both upper (URTIs) and lower (LRTIs) respiratory tract infections were included. URTIs were defined as coryza, pharyngitis, sinusitis, or cough with a clear chest CT-scan or X-ray. LRTIs were defined as exacerbations of chronic obstructive pulmonary disease following Anthonisen’s criteria or acute pneumonia with hypoxia and a new infiltrate on chest X-ray. The reference lists of the eligible articles and published meta-analyses were manually searched to identify undetected eligible studies. All identified articles were downloaded and read for duplicates exclusion and eligibility assessment. Searches and data extraction were done alongside by two investigators (NK and VL). Conflicts over inclusion were discussed until agreement with a third investigator (MD). For each included trial, the following information were extracted from published reports: authors, publication year, study design, inclusion period, and country, patients median age, viral diagnosis technique, diagnosis samples, number of patients diagnosed with hMPV-infections, hMPV-URTIs, hMPV-LRTIs, number of ICU admissions, ICU and hospital mortality rates, and viral, bacterial, and fungal coinfections rates. Underlying clinical characteristics of patients were also extracted: heart or respiratory comorbidities, and immunosuppression state defined as hematological malignancies, solid tumors, solid organ transplantation, human immunodeficiency virus infection, immunosuppressive treatment.

#### Data analysis

The primary endpoint was the LRTIs rate among hMPV infected patients. Secondary endpoints were ICU admission and pooled hospital and ICU mortality rates among hMPV infected patients. Overall untransformed LRTIs, ICU admission, hospital and ICU mortality rates were assessed, and their 95% confidence interval was calculated using both fixed and random-effects models. Publication bias was assessed by visually inspecting the funnel plot. Cochran’s *χ*^2^ test and *I*^2^ test for heterogeneity were used to assess inter-study heterogeneity [6]. The *χ*^2^ test assesses whether observed differences among results are compatible with chance alone and the *I*^2^ describes the percentage of the variability in effect estimates that results from heterogeneity rather than from the sampling error. An *I*^2^ test for heterogeneity above 0.25 was considered to indicate moderate heterogeneity. Statistically significant heterogeneity was considered present at *χ*^2^ P < 0.10 and *I*^2^ > 50%. All effect sizes with a *p* < 0.05 were considered significant. Tests were two-sided. In way to explain heterogeneity, a metaregression was performed. Last, three sensitivity analyses were performed in ways to assess impact of publication bias in the observed results using, namely, trim and fill method [7, 8], Copas method [9], and Outcome Reporting bias method [10]. All analyses were carried out with software R, version 3.6.2. The ‘Meta’, the ‘metasens’, and ‘metafor’ packages were used to produce forest plots and to run metaregression and sensitivity analyses. Quality assessment was conducted using the Newcastle–Ottawa quality assessment scales for case–control and cohort studies [11]. It is composed of three categories: Selection, Comparability and Outcome. A study can be awarded a maximum of nine stars/points. Comparability category was assessed regarding respiratory symptoms. The study protocol was registered at the National Institute for Health Research International Prospective Register of Systematic Reviews under the number CRD42018106617. The results are reported following the PRISMA checklist (Additional file 1: Appendix 2). 

## Results

### Cohort study and case–control analysis

**Fig. 1:**
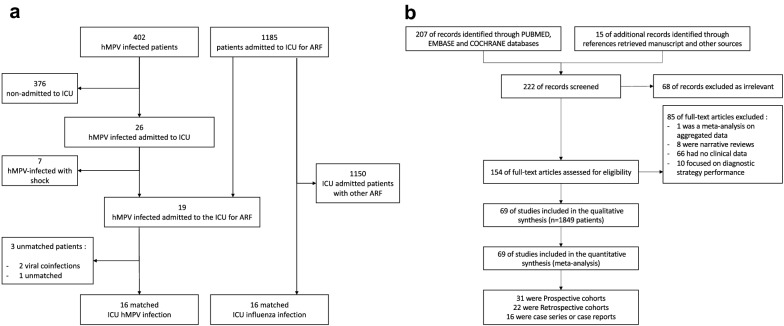
Flow charts. **a** Flow chart of the cohort study. **b** Flow chart of the systematic review. *ARF* acute respiratory failure, *ICU* intensive care unit, *hMPV* human metapneumovirus

**Fig. 2:**
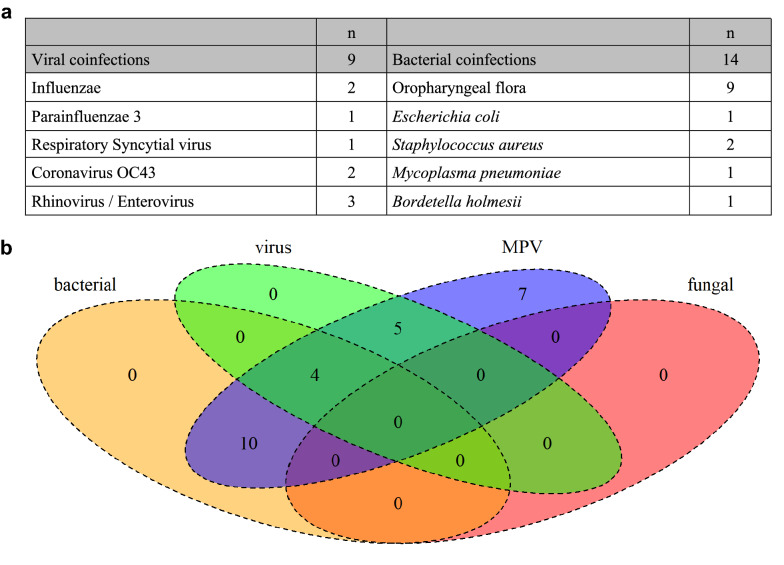
Coinfections in the hMPV-infection cohort. **a** Bacterial and viral identifications. **b** Venn diagram presenting the distribution of coinfections. MPV: human metapneumovirus

Within the study period, 402 patients had at least one upper respiratory tract sample positive for hMPV. Among them, 26/402 (6.5%) were admitted to ICU and included in the cohort among whom 19/26 (73.1%) for acute respiratory failure with oxygen over 6L/min. hMPV infection accounted for 1.6% (19/1185) of all patients admitted to the ICU with acute respiratory failure within the same period (Fig. 1). hMPV was mostly identified (23/26) during the winter season (December to April). All hMPV infections were community acquired. Among the hMPV cohort, 15 patients (57.6%) were male with a median age of 66 (56–74) years. Twenty-four (92%) patients were immunocompromised, including 21 (81%) patients with hematological malignancy among whom 17 (65%) patients received chemotherapy or an immunotherapy in the last year. Among other patients, one patient had newly diagnosed multiple myeloma, one patient received substitution for a hypogammaglobulinemia related to post-autologous hematopoietic stem cell transplantation and two were allogeneic stem cell transplanted patients. The other three immunocompromised patients were uncontrolled HIV infection (1 patient), metastatic melanoma receiving anti-PD1 therapy (1 patient), and severe Chronic Obstructive Pulmonary Disease treated with systemic steroid (1 patient). The last two patients had no defined immunosuppression, but one presented chronic cardiac failure and diabetes mellitus. Length of symptoms before diagnosis was 7 (IQR 2–11) days. Symptoms included fever (88.5%), dyspnea (92.3%), and cough (73.1%). Lymphopenia was frequently observed (*n* = 19, 73.1%), whereas neutropenia occurred only in 7 (26.9%) patients. Chest X-rays was performed for 25 patients (96.2%). The main radiologic pattern was interstitial infiltration (*n* = 20, 80.0%), mostly bilateral and diffuse (*n* = 17, 80.0%) with pleural effusion (*n* = 5, 20.0%). Chest X-ray was normal for 4 patients (16.0%). A thoracic CT-scan was performed in 14 patients (53.8%). None of the CT-scans were normal. The main pattern was ground glass opacities (*n* = 10; 66.8%) associated with crazy paving (*n* = 3, 20.0%). Alveolar condensations occurred in 9 (60%) patients, as well as nodular lesions (n = 9; 60%). For three patients, hMPV diagnosis was confirmed on lower respiratory tract samples. In 2 patients the nasopharyngeal respiratory panel also detected bacteria: *Mycoplasma pneumoniae* (*n* = 1) and *Bordetella holmesii* (*n* = 1). In 14 patients a respiratory tract sample was taken for bacteriological analysis within the first 3 days of ICU admission (10 spontaneous sputum samples, 2 bronchoalveolar lavages, 1 distal protected aspirate and 1 bronchial aspiration). Among them only 5 patients did not receive any antibiotic before the sample. Bacteriological analysis identified *Escherichia coli* (*n* = 1), *Staphylococcus aureus* (*n* = 2), and oropharyngeal flora with a quantification higher than 10^7^ colony-forming units (*n* = 9). In total, bacterial coinfections were detected in 11 (42%) patients, viral coinfections in 5 (19%) and both viral and bacterial coinfections in 3 (12%) (Fig. 2). Median SAPS2 score at admission was 41 (IQR 37–58). Ten patients (38.5%) required mechanical ventilation. Among them, 5 met ARDS criteria with a median P/F ratio of 125 (110–128) classifying them as moderate [12] and none required ECMO-support. Vasopressors were required for 11 (42.3%) patients and 4 (15.4%) patients received renal replacement therapy during ICU stay. Neither antiviral treatment nor intravenous immunoglobulin was prescribed for hMPV infection. All patients received broad-spectrum antibiotic therapy for a median of 11 (IQR 8–15) days. All initial antibiotic therapies were appropriate to the bacterial identifications. No patient received any corticosteroid treatment for the management of ARDS. ICU and hospital length of stay were, respectively, 5 (IQR 3–8) days and 17 (IQR 9–27) days. ICU and hospital mortality rates were 23.1% and 30.8%, respectively (Additional file 1: Figure S1). In univariate analysis, factors associated with hospital mortality were SAPS2 score (*p* = 0.019), need for invasive mechanical ventilation (*p* = 0.03), vasopressors (*p* < 0.001), and renal replacement therapy (*p* = 0.008). Clinical, biological, and imaging findings are summarized in Table 1. The pairing process identified 16 control patients admitted to the ICU with influenza infection and matched for age, admission year and underlying hematological malignancy (Fig. 1). Comparison between matched and unmatched patients did not recall any statistically significant difference. Although clinical and biological presentations were not different between the hMPV and the influenza groups, length of symptoms before diagnosis was longer for hMPV infection with 9 (IQR 6–14) vs 3 (IQR 2–4) days (*p* < 0.001). Hospital mortality rate seems to stay lower than for influenza infected patients (18.6% vs 56%, *p* = 0.03). The result of the case–control analysis is also presented in Table 1.

**Table 1:** Clinical, biological, and radiological characteristics of patients admitted to ICU for hMPV infection and results of the univariate analysis comparing influenza and hMPV infected matched patients

Characteristics (n, %) or median (IQR)	hMPV infections Total (n=26)	hMPV infections Unmatched (n=10)	hMPV infections Matched (n=16)	Influenza infections (n=16)	P*
Age	66 (56–74)	62 (42–66)	70 (58–76)	69 (54–75)	0.66
Gender (male)	15 (57.6)	6 (60.0)	9 (56.3)	11 (68.8)	0.46
Hematological malignancy					
Multiple myeloma	8 (30.8)	4 (40.0)	4 (25)	4 (25)	
Lymphoma	7 (26.9)	3 (30.0)	4 (25)	5 (31.3)	
Myeloproliferative disorder	3 (11.5)	2 (20.0)	1 (6.3)	1 (6.3)	0.84
Acute leukemia	2 (7.7)	1 (10.0)	1 (6.3)	1 (6.3)	
Myelodysplastic syndrome	1 (3.8)	0 (0)	1 (6.3)	0 (0)	
Other comorbidities					
Diabetes mellitus	7 (26.9)	0 (0)	7 (43.8)	7 (43.8)	1
HIV infection	3 (11.5)	2 (20.0)	1 (6.3)	1 (6.3)	1
Chronic respiratory failure	6 (23.1)	1 (10.0)	5 (31.3)	6 (37.5)	0.70
Solid Cancer	3 (11.5)	1 (10.0)	2 (12.5)	0 (0)	0.14
Steroids within the last 3 months	11 (42.3)	5 (50)	6 (37.5)	4 (25)	0.70
Symptoms					
SAPS 2 score	41 (37–58)	46 (40–58)	40 (36–60)	37 (30–47)	0.44
Fever (>38°C)	23 (88.5)	8 (80.0)	15 (93.8)	14 (87.5)	0.50
Shivers	9 (34.6)	3 (30.0)	6 (37.5)	4 (25)	0.40
Flu-like syndrome	5 (19.2)	1 (10.0)	4 (25)	5 (31.3)	0.70
ENT syndrome	8 (30.8)	2 (20.0)	6 (37.5)	3 (18.8)	0.20
Cough	19 (73.1)	6 (60.0)	13 (81.3)	15 (93.8)	0.30
Sputum	9 (34.6)	2 (20.0)	7 (43.8)	6 (37.5)	0.70
Dyspnea	24 (92.3)	8 (80.0)	16 (100)	15 (93.8)	0.30
Wheezing	4 (15.4)	2 (20.0)	2 (12.5)	5 (31.3)	0.20
Thoracic pain	1 (3.8)	0 (0.0)	1 (6.3)	2 (12.5)	0.50
Digestive symptoms	7 (26.9)	3 (30.0)	4 (25)	2 (12.5)	0.40
Biology characteristics					
Neutropenia	7 (26.9)	4 (40.0)	3 (18.8)	0 (0)	0.07
Lymphopenia	19 (73.1)	9 (90.0)	10 (62.5)	10 (62.5)	1
Chest X ray performed	25 (96.2)	10 (100)	15 (93.8)	16 (100)	
Normal	4 (16.0) **	2 (20)	2 (13.3)**	2 (12.5)	1
Alveolar syndrome Interstitial syndrome	6 (24.0) ** 20 (80.0) **	2 (20) 6 (60)	4 (26.7)** 14 (93.3)**	4 (25) 8 (50)	1 0.25
Four quadrants involved	12 (48.0) **	2 (20)	10 (66.7)**	3.(18.8)	0.04
Pleural effusion	5 (20.0) **	3 (30)	2 (13.3)**	0 (0)	0.59
Ct scan performed	14 (53.8)	6 (60)	8 (50)	5 (31.3)	
Ground glass opacities	10 (66.8) **	4 (66.7)**	6 (75)**	3 (60)**	0.04
Alveolar consolidation	9 (60.0) **	4 (66.7)**	5 (62.5)**	3 (60)**	0.72
Septal thickening	3 (20.0) **	2 (33.3)**	1 (12.5)**	2 (40)**	0.76
Nodular lesions	9 (60.0) **	2 (33.3)**	7 (87.5)**	5 (100)**	0.91
Crazy paving	3 (20.0) **	2 (33.3)**	1 (12.5)**	2 (40)**	0.76
Bronchial lesions	2 (13.3) **	0 (0)**	2 (25)**	4 (80)**	0.04
Coinfections					
Viral	8 (30.8)	8 (80)	0 (0)	3 (18.8)	–
Bacterial	14 (53.8)	5 (50)	9 (56.3)	3 (18.8)	0.07
ICU management and outcomes					
Symptoms duration before ICU admission	7 (2–11)	2 (1–7)	9 (6–15)	3 (2–5)	< 0.001
Mechanical ventilation	10 (38.5)	4 (40)	6 (37.5)	7 (43.8)	0.72
ARDS	5 (19.2)	2 (20)	3 (18.8)	5 (31.2)	0.68
Vasopressor support	11 (42.3)	7 (70)	4 (25)	9 (56.3)	0.07
Renal replacement therapy	4 (15.4)	2 (20)	2 (12.5)	4 (25)	0.36
Antibiotic duration	11 (8–15)	11 (8–13)	8 (2–18)	8 (7–14)	0.85
Hospital length of stay (days)	17 (9–27)	18 (11–33)	17 (8–25)	10 (7–21)	0.16
ICU length of stay (days)	5 (3–8)	6 (3–8)	7 (5–11)	5 (3–12)	0.95
Hospital mortality	8 (30.8)	5 (50)	3 (18.6)	9 (56)	0.03

*ENT* Ear, Nose and Throat, *ICU* Intensive care unit, *HIV* Human immunodeficiency virus, *hMPV* Human metapneumovirus, *LRTIs* Low respiratory tract infections, *SAPS II* Simplified Acute Physiology Score

^*^Univariate analysis compares the hMPV and the influenza infected matched groups

^**^Percentages are calculated considering the actual number of exams performed

### Systematic review and meta-analyses

A total of 224 citations were screened, 156 articles were fully analyzed and 69 were ultimately included (Fig. 1). Most of the included studies were single-center studies, with a median inclusion year of 2010 (IQR 2008–2012) and a median number of hMPV infected patients of 10 (IQR 3–27). Median age was 59 (IQR 48–65) years. Most of the analyzed studies included only immunocompromised patients with a median proportion of 100% (IQR 41–100). Patients with hematological malignancies and hematopoietic stem cell transplanted patients represented, respectively, of 52% (IQR 0–100) and 0% (IQR 0–18). Coinfections whether viral, bacterial, or fungal was reported in 11% (IQR 0–33) (Additional file 1: Table S1). Less than one third of studies had a quality as assessed by the Newcastle–Ottawa scale of seven points or above (Additional file 1: Figure S2).

**Fig. 3:**
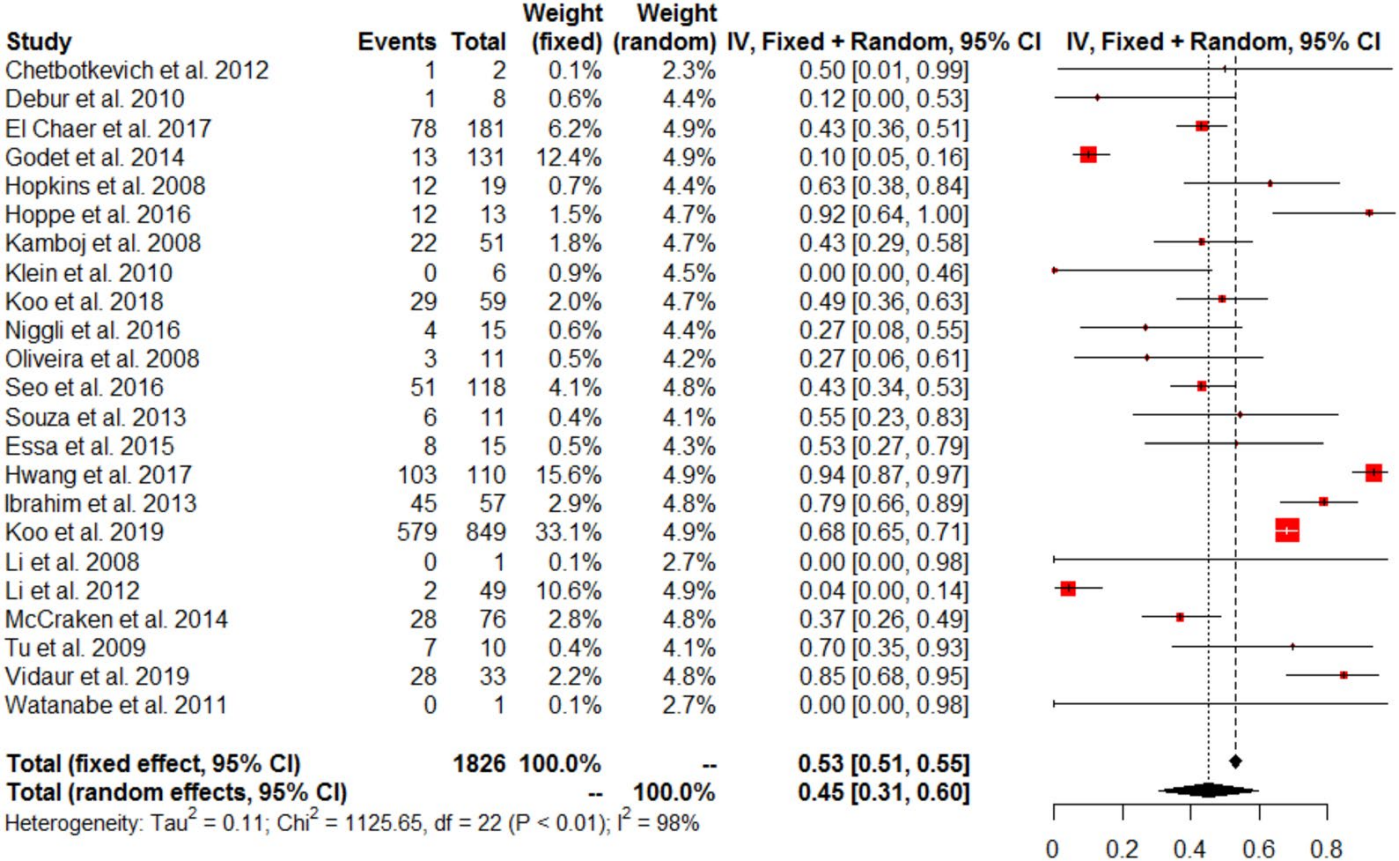
Forest-plot presenting the estimated rate of LRTIs in adults with hMPV infection (URTI and LRTI)

**Fig. 4:**
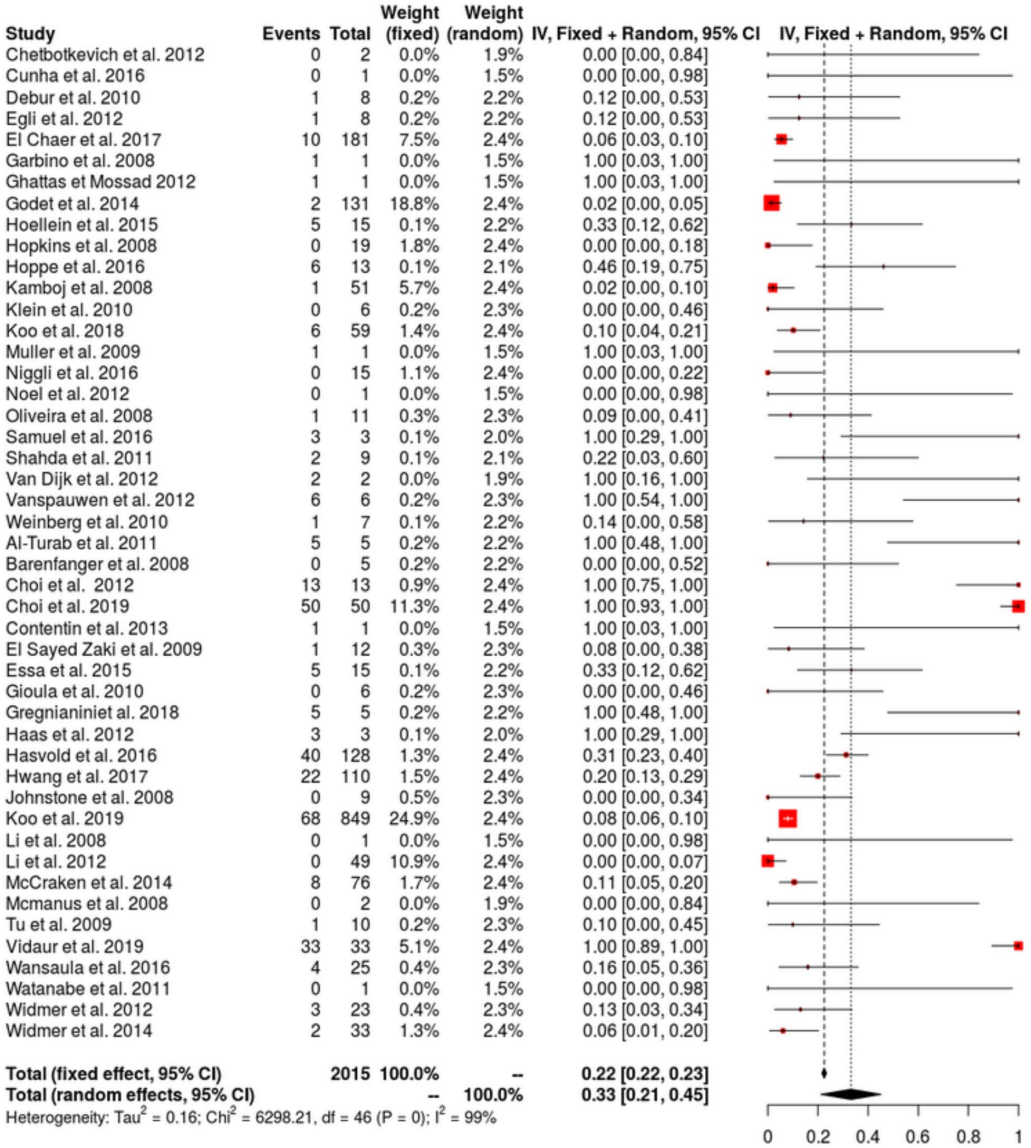
Forest-plot presenting the estimated rate of ICU admission in adults with hMPV infection (URTI and LRTI)

**Fig. 5:**
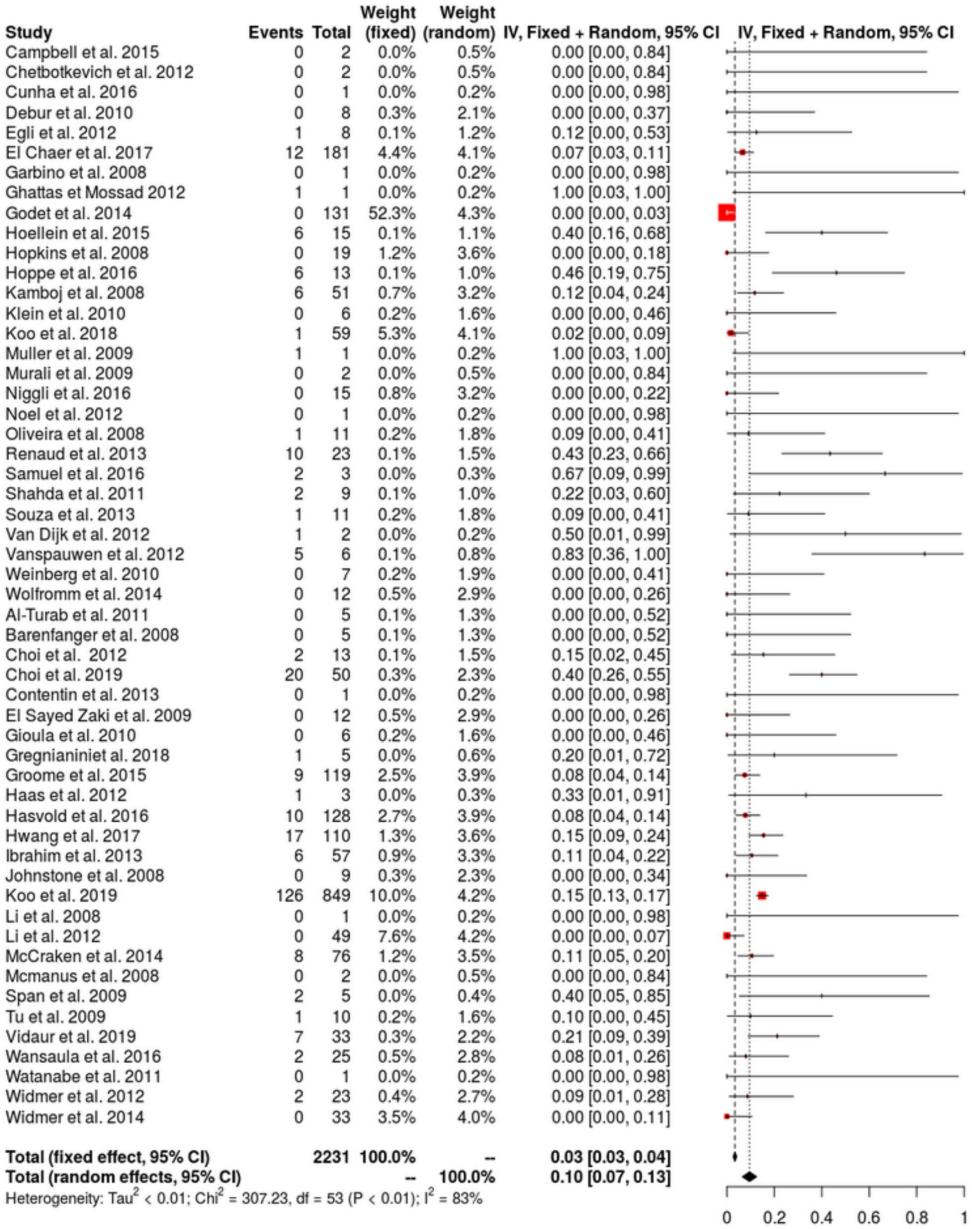
Forest-plot presenting the estimated mortality rate in adults with hMPV infection (URTI and LRTI)

Among hMPV infected patients, LRTIs rate was 45% (IQR 31–60, *I*^2^ = 98%) (23 studies, 1826 patients: Fig. 3). No publication bias was detected (Additional file 1: Figure S3a). LRTIs rate increased in recent publications (Additional file 1: Figure S4). Among hMPV infected patients, ICU admission was higher than in our cohort with 33% (IQR 21–45, *I*^2^ = 99%) (47 studies, 2015 patients: Fig. 4). No publication bias was detected (Additional file 1: Figure S3b). Pooled hospital mortality rate among hMPV infected patients was 10% (IQR 7–13, I^2^ = 83%) (47 studies, 2015 patients: Fig. 5) with a detected publication bias (Additional file 1: Figure S3c; *p* value < 0.001). Both publication year (Additional file 1: Figure S5a) and proportion of patients with underlying malignancy (Additional file 1: Figure S5b) were associated with increased mortality rate. ICU mortality rate was 23% (95% CI 12–34%; *I*^2^ = 65%) (5 studies, 158 patients). Sensitivity analyses with the 4 methods (Fill and trim method, Copas’ method, Outcome-reported bias method and sensitivity analysis excluding pediatric populations) for the 3 main analyses are reported in the Additional file 1: Appendix with relatively similar results (Additional file 1: Table S2).

## Discussion

This study was an exploratory study to question the pathogenicity of hMPV. Therefore, we conducted a comprehensive assessment combining a retrospective cohort study with a case–control analysis, and a systematic review of the literature. This systematic review was the first to comprehensively compile all the studies reporting hMPV upper and lower respiratory tract infections in adults.

First, our data reported great similarities in the clinical, biological, and radiological presentations between hMPV and influenza infections. Second, the meta-analysis revealed high rates of LRTIs (45%) and ICU admission (33%) among all hMPV infected patients. It could be the result of a reporting bias with an under-representation of hMPV-URTIs as they mainly concern outpatients with flu-like syndrome, and over-representation of hMPV-LRTIs, with the democratization of multiplex PCR respiratory panels in patients with dyspnea and fever. This might also explain the difference between the admission rate in the cohort study (6.5%) and the meta-analysis. Third, the meta-analysis observed a 10% hospital mortality rate among all hMPV infections, close to the 9% mortality rate described in influenza infected adults admitted to the ICU in 2021–2022 [13]. Finally, the meta-analysis suggested an increased mortality in hMPV infected patients with an underlying hematological malignancy. Consistently, the hospital mortality rate was higher (30.8%) in our cohort study including mostly immunocompromised patients. This hospital mortality rate remained close to the 38% hospital mortality rate for influenza infected immunocompromised patients admitted to the ICU [14]. Unfortunately, patients included in the observational study had mixed immunological profiles. Thus, no specific kind of immunosuppression could be related to hMPV infection. Those results raise the question of respiratory isolation policies for hematological patients due to the severity of disease and lack of specific treatment for hMPV.

Coinfection was present in 73% of the hMPV infected patients, which is much higher than the 11% found in the meta-analysis. The rate of bacterial coinfection particularly (53.8%) was closest to the rate of Koo *and coworkers*’ study on solid organ transplant recipients (48%) [15], and higher than the 24% or 25% coinfection rates observed in the other ICU cohorts by Vidaur *and coworkers* [16], or Choi *and coworkers* [17]. Interestingly, in the cohort study, the bacterial coinfection rate was high and duration between the symptoms onset and admission to the ICU was longer than in the influenza group (3 vs 8 days). These results could be interpreted in two ways. First, in immunocompromised patients, hMPV might be a contributing factor for bacterial infection with commensal germs of the airway microbiota. Viral-bacterial coinfections have been described mainly in the context of influenza but can be described with other respiratory viruses [18]. In the context of hMPV, the mechanisms identified in the literature could be: (i) the alteration of the host upper respiratory tract microbiota [19]; (ii) the alteration of the mucociliary clearance promoting bacterial stagnation [20]; (iii) the increased expression of bacterial membrane receptors promoting bacterial adhesion [21]; (iv) the destruction of the epithelial cells and relaxation of the tight junctions increasing bacterial translocation; (v) and finally, the alteration of host innate immune response [22][22]. On the other hand, in those immunocompromised patients hMPV could only reflect the degree of immunosuppression, the real pathogen being bacteria. As a matter of fact, viral detection in upper respiratory tract has been associated with mortality in a recent study including hematological patients[24].

This work had several limitations. First, diagnosis was made on upper respiratory tract samples. Therefore, some of those hMPV infections may be URTIs in severely ill patients admitted to ICU for another reason. However, even if mismatched have been described between upper and lower respiratory tract samples for the detection of hMPV the pairwise study present only small sample size [25]. Moreover, in immunocompromised patients with acute respiratory failure the broncho-alveolar lavage is no longer the cornerstone of the diagnosis strategy since 2008 [26]. A more recent cohort study even demonstrated a higher mortality rate in the invasive strategy compared to non-invasive strategy in such patients [27]. Therefore, most diagnosis strategies included only non-invasive strategies in such patients. Second limitation, the cohort was retrospective and monocentric with a small sample size especially regarding the case–control analysis and included a vast majority of immunocompromised patients. Therefore, studies including more centers and patients are warranted to describe hMPV infection in non-immunocompromised patients. Moreover, collection bias regarding clinical signs, radiological evaluations and diagnosis of bacterial coinfection cannot be denied. Indeed, respiratory samples for bacterial analysis were not performed in all patients, and most of them received antibiotics before. Therefore, the bacterial coinfection rate could be under-estimated. Finally, we could not analyze in this study the impact of steroids. None of patient received steroid for ARDS management and only 11/26 received steroids before ICU admission with no impact on mortality rate in this small sample. In addition, impact of initial antibiotic could not be analyzed, because all patients received adequate initial antibiotic when infection was documented**.** Nevertheless, this was one of the most important studies describing those patients in the literature. In the systematic review, despite an attempt to collect all published studies on the subject, most studies collected were retrospective, non-randomized, monocentric, with small sample sizes, leading to a high heterogeneity between studies. Moreover, the analysis of the Funnel-plot concerning mortality had a publication bias related to the lower publication rate on ambulatory studies and an over-estimation of studies including immunocompromised and hospitalized patients. Regarding this heterogeneity, more cohort studies to assess attributable morbidity and mortality are warranted.

In conclusion, this work provides information regarding hMPV characteristics in immunocompromised patients. Considering the high rate of bacterial coinfections, microbiological screening should be performed carefully, and antibiotic treatment should be always discussed in patient with ARF.

## Supplementary Information

**Additional file 1: Appendix 1.** Search strategy. **Appendix 2.** PRISMA checklist. **Figure S1.** Kaplan–Meier survival curve until 6 months in the hMPV cohort. **Figure S2.** Results of the Newcastle–Ottawa quality assessment for all trials. **Figure S3.** Forrest plots of the 3 analyses of the systematic review: a Prevalence of Low Respiratory Tract Infections. b ICU admission rate. c Mortality. **Figure S4.** Bubble plot presenting the correlation between the proportion of low respiratory tract infections and the publication year. **Table S1.** Characteristics of all studies included in the systematic review analysis. **Table S2.** Sensitivity analysis with the 4 methods: Fill and trim method, Copas’ method, Outcome-reported bias method and sensitivity analysis excluding pediatric populations

## Data Availability

Data are available from the corresponding authors for reasonable request.

## Abbreviations

ECMO: Extra-corporeal membrane-oxygenation
hMPV: Human metapneumovirus
HIV: Human immunodeficiency virus
ICU: Intensive care unit
LRTI: Low respiratory tract infection
PCR: Polymerase chain reaction
SARS-CoV-2: Severe acute respiratory syndrome coronavirus 2
URTI: Upper respiratory tract infection
