# Supplementary material for: Clinical significance of human metapneumovirus detection in critically ill adults with lower respiratory tract infections
Source: Ann Intensive Care. 2023 Mar 20;13:21. doi: 10.1186/s13613-023-01117-w (PMC10026215; doi:10.1186/s13613-023-01117-w)
Supplement: Supplementary file 1 — Additional file 1: Appendix 1. Search strategy. Appendix 2. PRISMA checklist. Figure S1. Kaplan–Meier survival curve until 6 months in the hMPV cohort. Figure S2. Results of the Newcastle–Ottawa quality assessment for all trials. Figure S3. Forrest plots of the 3 analyses of the systematic review: a Prevalence of Low Respiratory Tract Infections. b ICU admission rate. c Mortality. Figure S4. Bubble plot presenting the correlation between the proportion of low respiratory tract infections and the publication year. Table S1. Characteristics of all studies included in the systematic review analysis. Table S2. Sensitivity analysis with the 4 methods: Fill and trim method, Copas’ method, Outcome-reported bias method and sensitivity analysis excluding pediatric populations [file 13613_2023_1117_MOESM1_ESM.docx]

**Online Supplementary Material**

**Clinical significance of human metapneumovirus detection in critically ill adults with lower respiratory tract infections**

Natacha Kapandji^1^ MD, Michael Darmon^1^ MD-PhD, Sandrine Valade^1^ MD, Maud Salmona^2^ MD, Jérôme Legoff^2^ MD-PhD, Lara Zafrani^1^ MD-PhD, Elie Azoulay^1^ MD-PhD, Virginie Lemiale^1^ MD.

1. Intensive Care Unit, Saint Louis Academic Hospital, Paris, France

^2.^ Virology department, Saint Louis hospital, Paris, France

**Corresponding author:** Dr Natacha Kapandji

Medical ICU, Saint Louis hospital, APHP

1 Avenue Claude Vellefaux, 75010 PARIS, France

Phone : 0033 6 67 33 92 14

Mail : natacha.kapandji@aphp.fr

**Appendix 1: Search strategy**

**PUBMED**

1. (human metapneumovirus [MeSH Terms]) AND (adult [MeSH Terms])
2. 1 And filter: January 1, 2008 and August 31, 2019
3. 2 And filter: English
4. 3 and filter: Humans
5. 4 and filter: Adults +19 years
6. (human metapneumovirus [MeSH Terms]) NOT (children [MeSH Terms])
7. 6 and 2,3,4,5

(155 results)

**EMBASE**

1. #1 ‘human metapneumovirus’:ti, ab, kw AND [english]/lim AND [adult]/lim AND [2008-2019]py AND [humans]/lim
2. #2 ‘human metapneumovirus infection’:ti, ab, kw AND [english]/lim AND [adult]/lim AND [2008-2019]py AND [humans]/lim
3. #3 ‘human metapneumoviruses’:ti, ab, kw AND [english]/lim AND [adult]/lim AND [2008-2019]py AND [humans]/lim
4. #4 ‘human metapneumoviruses infection’:ti, ab, kw AND [english]/lim AND [adult]/lim AND [2008-2019]py AND [humans]/lim

(423 results)

**COCHRANE LIBRARY**

1. #1 MeSH descriptor: [Human metapneumovirus] explode all trees

(2 results)

**Appendix 2: PRISMA checklist**

| **Section/topic** | **#** | **Checklist item** | **Reported on page #** |
| --- | --- | --- | --- |
| **TITLE** | | |  |
| Title | 1 | Identify the report as a systematic review, meta-analysis, or both. | 1 |
| **ABSTRACT** | | |  |
| Structured summary | 2 | Provide a structured summary including, as applicable: background; objectives; data sources; study eligibility criteria, participants, and interventions; study appraisal and synthesis methods; results; limitations; conclusions and implications of key findings; systematic review registration number. | 2 |
| **INTRODUCTION** | | |  |
| Rationale | 3 | Describe the rationale for the review in the context of what is already known. | 3 |
| Objectives | 4 | Provide an explicit statement of questions being addressed with reference to participants, interventions, comparisons, outcomes, and study design (PICOS). | 3 |
| **METHODS** | | |  |
| Protocol and registration | 5 | Indicate if a review protocol exists, if and where it can be accessed (e.g., Web address), and, if available, provide registration information including registration number. | 5 and 7 |
| Eligibility criteria | 6 | Specify study characteristics (e.g., PICOS, length of follow-up) and report characteristics (e.g., years considered, language, publication status) used as criteria for eligibility, giving rationale. | 5-6 |
| Information sources | 7 | Describe all information sources (e.g., databases with dates of coverage, contact with study authors to identify additional studies) in the search and date last searched. | 5-6 |
| Search | 8 | Present full electronic search strategy for at least one database, including any limits used, such that it could be repeated. | Appendix 1 |
| Study selection | 9 | State the process for selecting studies (i.e., screening, eligibility, included in systematic review, and, if applicable, included in the meta-analysis). | 5-6 |
| Data collection process | 10 | Describe method of data extraction from reports (e.g., piloted forms, independently, in duplicate) and any processes for obtaining and confirming data from investigators. | 5-6 |
| Data items | 11 | List and define all variables for which data were sought (e.g., PICOS, funding sources) and any assumptions and simplifications made. | 5-6 |
| Risk of bias in individual studies | 12 | Describe methods used for assessing risk of bias of individual studies (including specification of whether this was done at the study or outcome level), and how this information is to be used in any data synthesis. | 7 |
| Summary measures | 13 | State the principal summary measures (e.g., risk ratio, difference in means). | 7 |
| Synthesis of results | 14 | Describe the methods of handling data and combining results of studies, if done, including measures of consistency (e.g., I^2^) for each meta-analysis. | 7 |

**Appendix 3: Supplemental Figures**

**Figure S1: Kaplan Meier survival curve until 6 months in the hMPV cohort**

**Figure S2: Results of the Newcastle-Ottawa quality assessment for all trials.**

Two quality assessment scales were used for cohort and case-control studies. The scales consist of 3 categories: selection, comparability and outcome or exposure depending on the type of study. Each study is granted with a maximum of 9 points and a minimum of 0. The histograms represent the distribution of studies included in each analysis.

**Figure S3: Forrest plots of the 3 analyses of the systematic review:**

1. Prevalence of Low Respiratory Tract Infections
2. ICU admission rate
3. Mortality

**Figure S4: Bubble plot presenting the correlation between the proportion of low respiratory tract infections and the publication year.**

**Figure S5: Bubble plot presenting the correlation between mortality rate and:**

1. Publication year
2. Proportion of patients presenting hematological malignancy

**Figure S1:
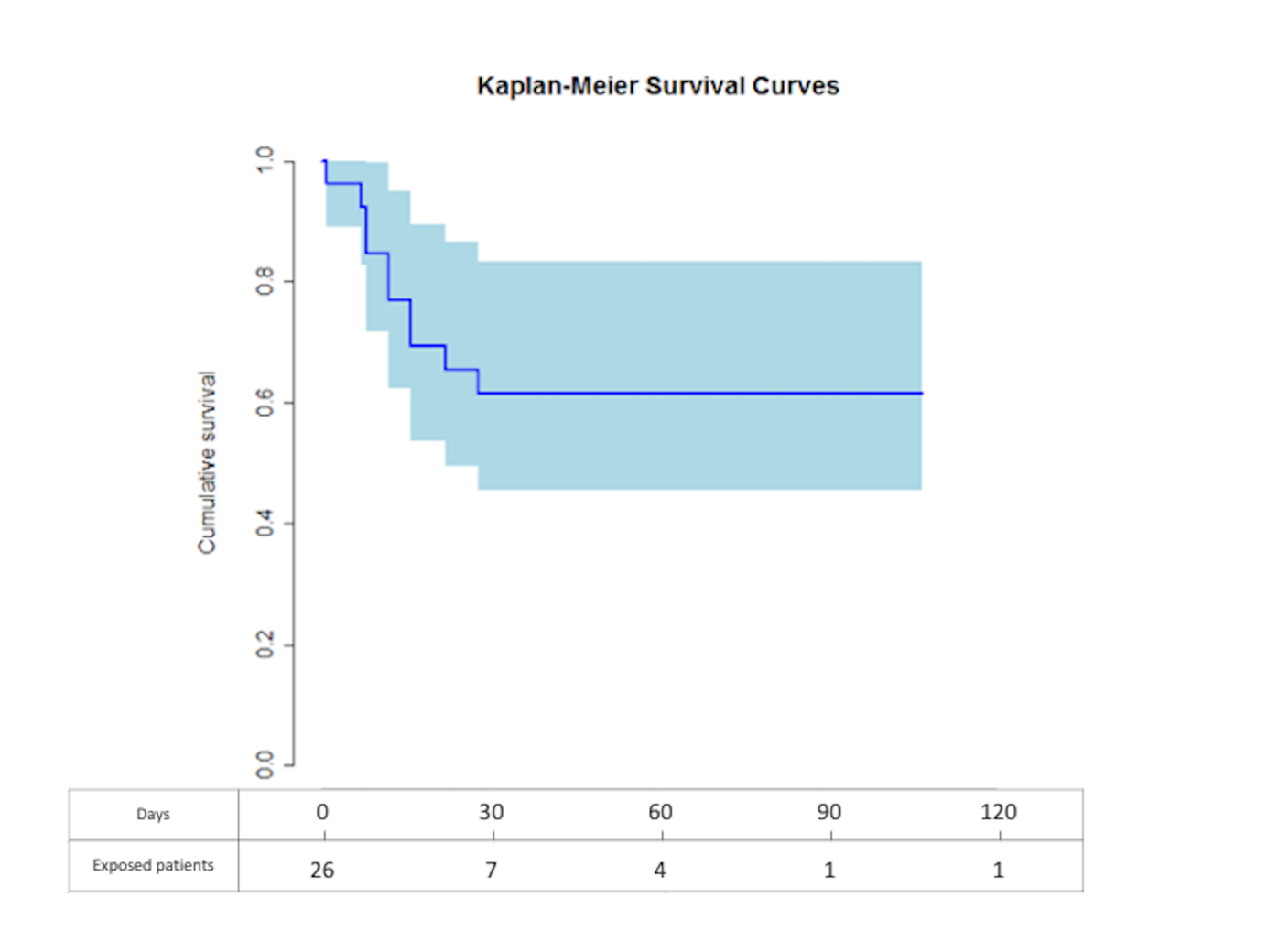
**

**
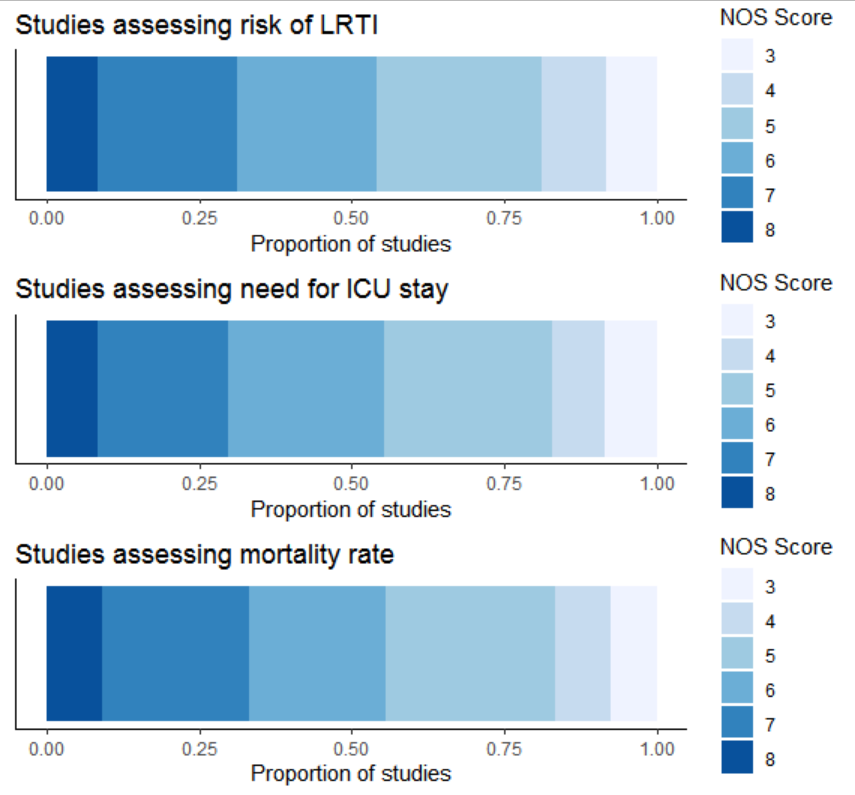
Figure S2:**

**Figure S3:**

**b.**

**c.**

**a.**

**
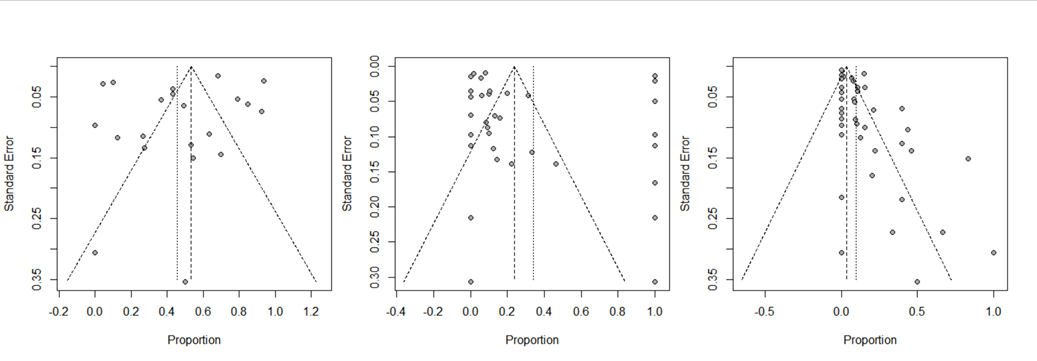
**

**Figure S4:**

**
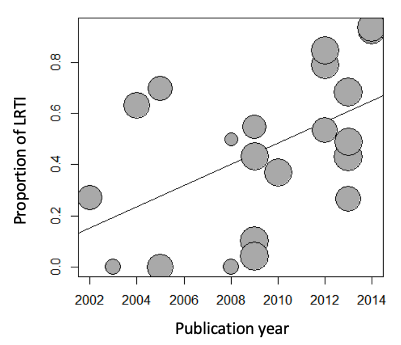
**

**Figure S5:**

**b.**

**a.**


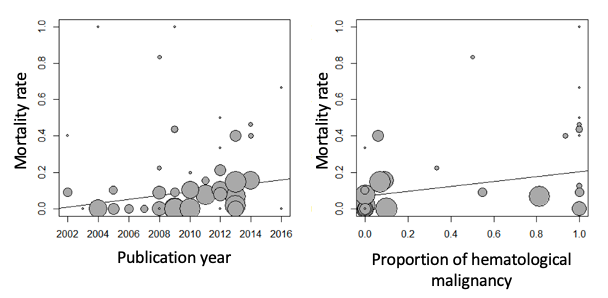


**Appendix 4: Supplemental tables**

**Table S1: Characteristics of all studies included in the systematic review analysis**

hMPV: human metapneumovirus; ICU: intensive care unit; IF: immunofluorescence; LRTIs: lower respiratory tract infections; MLPA: multiplex ligation-dependent probe amplification; NA: Not applicable; RT-PCR: reverse transcription and polymerase chain reaction

|  | Study type | Dates | Diagnostic methods | Trials Newcastle - Ottawa quality assessment scores | Number of hMPV (+) and tested patients (n/N) | Number of LRTIs (n) | Number of ICU admissions (n) | Number of hospital deaths  (n) | Number of coinfected patients  (n) |
| --- | --- | --- | --- | --- | --- | --- | --- | --- | --- |
| Al-Turab et al. (2011)^1^ | Prospective cohort | 2009 | RT-PCR | 4 | 5/63 | 5 | 5 | 0 | NA |
| Barenfanger et al. (2008)^2^ | Prospective cohort | 2006-07 | Immunofluorescence and viral culture | 4 | 5/161 | 5 | 0 | 0 | NA |
| Campbell et al. (2015)^3^ | Prospective cohort | 2005-10 | RT-PCR | 5 | 2/406 | NA | NA | 0 | NA |
| Chetbotkevich et al. (2012)^4^ | Prospective cohort | 2008-09 | RT-PCR | 5 | 2/48 | 1 | 0 | 0 | 1 |
| Choi et al. (2012)^5^ | Retrospective cohort | 2010-11 | Multiplex | 6 | 13/198 | 13 | 13 | 2 | 3 |
| Choi et al. (2019)^6^ | Prospective cohort | 2010-17 | Multiplex | 6 | 50/1556 | 50 | 50 | 20 | 15 |
| Contentin et al. (2013)^7^ | Case report | 2012 | Multiplex | 3 | 1/1 | 1 | 1 | 0 | 0 |
| Cunha et al. (2016)^8^ | Case report | 2016 | RT-PCR | 3 | 1/1 | 1 | 0 | 0 | 0 |
| Debur et al. (2010)^9^ | Retrospective cohort | 2000-02 / 2006-08 | RT-PCR | 6 | 8/189 | 1 | 1 | 0 | 1 |
| Djamin et al. (2015)^10^ | Retrospective cohort | 2010-13 | RT-PCR | 2 | 5/63 | 5 | NA | NA | 0 |
| Egli et al. (2012)^11^ | Prospective cohort | 2009-10 | Multiplex | 5 | 8/93 | 8 | 1 | 1 | 3 |
| El Chaer et al. (2017)^12^ | Retrospective cohort | 2012-15 | Multiplex | 7 | 181/NA | 78 | 10 | 12 | 7 |
| El Sayed Zaki et al. (2009)^13^ | Prospective cohort | 2007-09 | RT-PCR | 6 | 12/88 | 12 | 1 | 0 | 4 |
| Essa et al. (2015)^14^ | Prospective cohort | 2010-13 | Multiplex | 6 | 15/735 | 8 | 5 | NA | NA |
| Feng et al. (2014)^15^ | Prospective cohort | 2009-13 | RT-PCR | 5 | 165/7732 | 165 | NA | NA | NA |
| Gambarino et al. (2009)^16^ | Prospective cohort | 2008 | Multiplex | 4 | 13/219 | 13 | NA | NA | NA |
| Garbino et al. (2008)^17^ | Prospective cohort | 2003-06 | Multiplex | 6 | 1/55 | 1 | 1 | 0 | 0 |
| Ghattas and Mossad (2012)^18^ | Case report | 2009 | Immunofluorescence | 5 | 1/NA | 1 | 1 | 1 | 1 |
| Gioula et al. (2010)^19^ | Prospective cohort | 2005-08 | RT-PCR | 8 | 6/149 | NA | 0 | 0 | NA |
| Godet et al. (2014)^20^ | Case report | 2007-11 | Multiplex and immunofluorescence | 7 | 131/11267 | 13 | 2 | 0 | 2 |
| Gregnianini et al. (2018)^21^ | Prospective cohort | 2009-11 | RT-PCR | 5 | 5/NA | 5 | 5 | 1 | NA |
| Groome et al. (2015)^22^ | Prospective cohort | 2009-13 | RT-PCR | 8 | 119/6934 | 119 | NA | 9 | 49 |
| Guido et al. (2011)^23^ | Prospective cohort | 2008-09 | RT-PCR | 4 | 6/64 | NA | NA | NA | NA |
| Haas et al. (2012)^24^ | Case report | 2012 | RT-PCR | 4 | 3/3 | 3 | 3 | 1 | 0 |
| Hasvold et al. (2016)^25^ | Retrospective cohort | 2009-13 | RT-PCR and viral culture | 5 | 128/335 | NA | 40 | 10 | 9 |
| Hoellein et al. (2015)^26^ | Case report | 2014 | RT-PCR | 5 | 15/NA | 15 | 5 | 6 | 0 |
| Hopkins et al. (2008)^27^ | Prospective cohort | 2003-06 | RT-PCR and immunofluorescence | 8 | 19/89 | 12 | 0 | 0 | 17 |
| Hoppe et al. (2016)^28^ | Case report | 2014 | MLPA | 7 | 13/NA | 12 | 6 | 6 | 0 |
| Huijts et al. (2018)^29^ | Retrospective cohort | 2008-13 | RT-PCR | 4 | 36/1653 | 36 | NA | NA | NA |
| Hwang et al. (2017)^30^ | Retrospective cohort | 2012-16 | Multiplex | 7 | 110/110 | 103 | 22 | 17 | 41 |
| Ibrahim et al. (2013)^31^ | Case report | 2012 | RT-PCR | 7 | 57/163 | 45 | NA | 6 | NA |
| Johnstone et al. (2008)^32^ | Prospective cohort | 2004-06 | RT-PCR | 6 | 9/193 | 9 | 0 | 0 | 1 |
| Kamboj et al. (2008)^33^ | Retrospective cohort | 2005-07 | RT-PCR and immunofluorescence | 7 | 51/2125 | 22 | 1 | 6 | 3 |
| Klein et al. (2010)^34^ | Retrospective cohort | 2003-06 | Multiplex | 5 | 6/50 | 0 | 0 | 0 | 2 |
| Koo et al. (2018)^35^ | Retrospective cohort | 2010-16 | Multiplex | 7 | 59/NA | 29 | 6 | 1 | 23 |
| Koo et al. (2019)^36^ | Retrospective cohort | 2010-16 | Multiplex | 8 | 849/15311 | 579 | 68 | 126 | 126 |
| Kwon et al. (2012)^37^ | Retrospective cohort | 2009-11 | Multiplex | 2 | 1/147 | NA | NA | NA | NA |
| Li et al. (2008)^38^ | Case report | 2008 | Multiplex | 3 | 1/1 | 0 | 0 | 0 | 0 |
| Li et al. (2012)^39^ | Prospective cohort | 2008-10 | RT-PCR | 5 | 49/2936 | 2 | 0 | 0 | 6 |
| McCraken et al. (2014)^40^ | Prospective cohort | 2007-12 | Multiplex | 8 | 76/6288 | 28 | 8 | 8 | NA |
| McManus et al. (2008)^41^ | Prospective cohort | NA | RT-PCR | 4 | 2/136 | 2 | 0 | 0 | NA |
| Mikulska et al. (2014)^42^ | Prospective cohort | 2011 | Multiplex | 3 | 0/139 | NA | NA | NA | NA |
| Muller et al. (2009)^43^ | Retrospective cohort | 2003-04 | RT-PCR | 6 | 1/128 | 1 | 1 | 1 | 0 |
| Murali et al. (2009)^44^ | Retrospective cohort | 2006-07 | Multiplex | 4 | 2/70 | 2 | NA | 0 | 1 |
| Niggli et al. (2016)^45^ | Retrospective cohort | 2012-14 | Multiplex | 6 | 15/NA | 4 | 0 | 0 | 4 |
| Noel et al. (2012)^46^ | Case report | 2012 | Immunofluorescence | 3 | 1/1 | 1 | 0 | 0 | 0 |
| Oliveira et al. (2008)^47^ | Prospective cohort | 2001-03 | RT-PCR | 7 | 11/153 | 3 | 1 | 1 | 3 |
| Park et al. (2013)^48^ | Retrospective cohort | 2009-12 | Multiplex | 2 | 21/737 | NA | NA | NA | NA |
| Peyrani et al. (2012)^49^ | Prospective cohort | 2010-11 | Multiplex | 2 | 3/150 | 3 | NA | NA | NA |
| Renaud et al. (2013)^50^ | Retrospective cohort | 2006-11 | Multiplex and immunofluorescence | 7 | 23/NA | 23 | NA | 10 | 13 |
| Samuel et al. (2016)^51^ | Case report | 2016 | Multiplex | 5 | 3/NA | 3 | 3 | 2 | 0 |
| Seo et al. (2016)^52^ | Retrospective cohort | 2004-14 | Multiplex and immunofluorescence | 3 | 118/NA | 51 | NA | NA | 36 |
| Shahda et al. (2011)^53^ | Case report | 2008-09 | Multiplex | 5 | 9/NA | 9 | 2 | 2 | 3 |
| Souza et al. (2013)^54^ | Retrospective cohort | 2008-09 | RT-PCR | 7 | 11/75 | 6 | NA | 1 | 0 |
| Span et al. (2009)^55^ | Retrospective cohort | 1999-2006 | RT-PCR | 5 | 5/97 | 5 | NA | 2 | 0 |
| Syha et al. (2012)^56^ | Case report | 2011-12 | Multiplex | 2 | 10/NA | 10 | NA | NA | 0 |
| Tu et al. (2009)^57^ | Case report | 2005 | RT-PCR | 5 | 10/52 | 7 | 1 | 1 | 1 |
| Van Dijk et al. (2012)^58^ | Case report | 2012 | RT-PCR | 5 | 2/2 | 2 | 2 | 1 | 0 |
| Vanspauwen et al. (2012)^59^ | Prospective cohort | 2005-10 | RT-PCR | 5 | 6/290 | 6 | 6 | 5 | 0 |
| Vidaur et al. (2019)^60^ | Retrospective cohort | 2007-17 | RT-PCR | 6 | 33/1942 | 28 | 33 | 7 | 10 |
| Wansaula et al. (2016)^61^ | Case report | 2010-14 | Multiplex | 6 | 25/332 | 25 | 4 | 2 | 1 |
| Watanabe et al. (2011)^62^ | Prospective cohort | 2002-03 | RT-PCR | 6 | 1/365 | 0 | 0 | 0 | NA |
| Weinberg et al. (2010)^63^ | Prospective cohort | 2005-07 | RT-PCR | 7 | 7/112 | NA | 1 | 0 | 3 |
| Widmer et al. (2012)^64^ | Prospective cohort | 2006-09 | RT-PCR | 7 | 23/508 | 23 | 3 | 2 | 0 |
| Widmer et al. (2014)^65^ | Prospective cohort | 2009-10 | RT-PCR | 7 | 33/1248 | NA | 2 | 0 | 0 |
| Wolfromm et al. (2014)^66^ | Prospective cohort | 2006-10 | Multiplex | 6 | 12/378 | NA | NA | 0 | NA |
| Wong et al. (2012)^67^ | Retrospective cohort | 2008 | RT-PCR | 3 | 4/NA | 4 | NA | NA | 0 |
| Ye et al. (2017)^68^ | Prospective cohort | 2012-15 | RT-PCR | 3 | 27/967 | 27 | NA | NA | 7 |
| Yu et al. (2012)^69^ | Prospective cohort | 2010-11 | RT-PCR | 4 | 11/416 | 11 | NA | NA | 2 |

**Table S2: Sensitivity analysis with the 4 methods: Fill and trim method, Copas’ method, Outcome-reported bias method and sensitivity analysis excluding pediatric populations**

|  | Principal results | Fill and trim method | Copa’s method | Outcome-reporting-bias method | Sensitivity analysis excluding pediatric population |
| --- | --- | --- | --- | --- | --- |
| LRTIs rate | 45 [31 - 60%] | 70 [54 - 86%] | 46 [34 - 58%] | 49 [34 - 63%] | 45 [30 - 61%] |
| ICU admission rate | 33 [21 - 45%] | 31 [19 - 43%] | 29 [17 - 44%] | 33 [22 - 45%] | 34 [22 - 46%] |
| Mortality rate | 10 [7 - 13%] | 4 [1 - 7%] | 5 [3 - 7%] | 9 [6 - 12%] | 9 [6 - 13%] |

**References:**

1. Al‐Turab M, Chehadeh W, Al‐Mulla F, Al‐Nakib W. Human metapneumovirus in patients with respiratory tract infection in Kuwait. Journal of Medical Virology. 2011;83(10):1811‑7.

2. Barenfanger J, Mueller T, O’Brien J, Drake C, Lawhorn J. Prevalence of Human Metapneumovirus in Central Illinois in Patients Thought To Have Respiratory Viral Infection. Journal of Clinical Microbiology. 1 avr 2008;46(4):1489‑90.

3. Campbell AP, Guthrie KA, Englund JA, Farney RM, Minerich EL, Kuypers J, et al. Clinical Outcomes Associated With Respiratory Virus Detection Before Allogeneic Hematopoietic Stem Cell Transplant. Clin Infect Dis. 15 juill 2015;61(2):192‑202.

4. Chebotkevitch V. Abstracts accepted for publication only - R2355 -. Clinical Microbiology and Infection. 1 avr 2010;16:S635‑716.

5. Choi S-H, Hong S-B, Ko G-B, Lee Y, Park HJ, Park S-Y, et al. Viral Infection in Patients with Severe Pneumonia Requiring Intensive Care Unit Admission. American Journal of Respiratory and Critical Care Medicine. 15 août 2012;186(4):325‑32.

6. Choi S-H, Hong S-B, Huh JW, Jung J, Kim MJ, Chong YP, et al. Outcomes of severe human metapneumovirus-associated community-acquired pneumonia in adults. Journal of Clinical Virology. 1 août 2019;117:1‑4.

7. Contentin L, Guillon A, Garot D, Gaudy-Graffin C, Perrotin D. Acute respiratory distress syndrome secondary to human metapneumovirus infection in a young healthy adult. Intensive Care Medicine. mars 2013;39(3):533‑4.

8. Cunha BA, Irshad N, Connolly JJ. Adult human metapneumonovirus (hMPV) pneumonia mimicking Legionnaire’s disease. Heart & Lung. mai 2016;45(3):270‑2.

9. Debur MC, Vidal LR, Stroparo E, Nogueira MB, Almeida SM, Takahashi GA, et al. Human metapneumovirus infection in hematopoietic stem cell transplant recipients. Transplant Infectious Disease. 2010;12(2):173‑9.

10. Djamin RS, Uzun S, Snelders E, Kluytmans JJW, Hoogsteden HC, Aerts JGJV, et al. Occurrence of virus-induced COPD exacerbations during four seasons. Infectious Diseases. 1 févr 2015;47(2):96‑100.

11. Egli A, Bucher C, Dumoulin A, Stern M, Buser A, Bubendorf L, et al. Human metapneumovirus infection after allogeneic hematopoietic stem cell transplantation. Infection. 1 déc 2012;40(6):677‑84.

12. Chaer FE, Shah DP, Kmeid J, Ariza‐Heredia EJ, Hosing CM, Mulanovich VE, et al. Burden of human metapneumovirus infections in patients with cancer: Risk factors and outcomes. Cancer. 2017;123(12):2329‑37.

13. Zaki MES, Raafat D, El‐Metaal AA, Ismail M. Study of human metapneumovirus-associated lower respiratory tract infections in Egyptian adults. Microbiology and Immunology. 2009;53(11):603‑8.

14. Essa S, Owayed A, Altawalah H, Khadadah M, Behbehani N, Al-Nakib W. The Prevalence of Human Bocavirus, Human Coronavirus-NL63, Human Metapneumovirus, Human Polyomavirus KI and WU in Respiratory Tract Infections in Kuwait. MPP. 2015;24(4):382‑7.

15. Feng L, Li Z, Zhao S, Nair H, Lai S, Xu W, et al. Viral Etiologies of Hospitalized Acute Lower Respiratory Infection Patients in China, 2009-2013. PLOS ONE. 19 juin 2014;9(6):e99419.

16. Gambarino S, Mantovani S, Astegiano S, Libertucci D, Solidoro P, Baldi S, et al. Lower respiratory tract viral infections A in hospitalized adult patients. MINERVA MEDICA. 2009;100(5):8.

17. Garbino J, Inoubli S, Mossdorf E, Weber R, Tamm M, Soccal P, et al. Respiratory viruses in HIV-infected patients with suspected respiratory opportunistic infection: AIDS. mars 2008;22(6):701‑5.

18. Ghattas C, Mossad SB. Fatal human metapneumovirus and influenza B virus coinfection in an allogeneic hematopoietic stem cell transplant recipient. Transplant Infectious Disease. 2012;14(5):E41‑3.

19. Gioula G, Chatzidimitriou D, Melidou A, Exindari M, Kyriazopoulou-Dalaina V. Contribution of human metapneumovirus to influenza-like infections in North Greece, 2005-2008. Eurosurveillance. 4 mars 2010;15(9):19499.

20. Godet C, Le Goff J, Beby-Defaux A, Robin M, Raffoux E, Arnulf B, et al. Human metapneumovirus pneumonia in patients with hematological malignancies. Journal of Clinical Virology. 1 déc 2014;61(4):593‑6.

21. Gregianini TS, Seadi CF, Menegolla I, Martins LG, Ikuta N, Wolf JM, et al. Human metapneumovirus in Southern Brazil. Revista da Sociedade Brasileira de Medicina Tropical. févr 2018;51(1):30‑8.

22. Groome MJ, Moyes J, Cohen C, Walaza S, Tempia S, Pretorius M, et al. Human metapneumovirus-associated severe acute respiratory illness hospitalisation in HIV-infected and HIV-uninfected South African children and adults. Journal of Clinical Virology. 1 août 2015;69:125‑32.

23. Guido M, Quattrocchi M, Campa A, Zizza A, Grima P, Romano A, et al. Human metapneumovirus and human bocavirus associated with respiratory infection in Apulian population. Virology. août 2011;417(1):64‑70.

24. Haas LEM, de Rijk NX, Thijsen SFT. Human metapneumovirus infections on the ICU: a report of three cases. Ann Intensive Care. 19 juill 2012;2(1):30.

25. Hasvold J, Sjoding M, Pohl K, Cooke CR, Hyzy RC. The role of human metapneumovirus in the critically ill adult patient,. J Crit Care. févr 2016;31(1):233‑7.

26. Hoellein A, Hecker J, Hoffmann D, Göttle F, Protzer U, Peschel C, et al. Serious outbreak of human metapneumovirus in patients with hematologic malignancies. Leukemia & Lymphoma. 3 mars 2016;57(3):623‑7.

27. Hopkins P, McNeil K, Kermeen F, Musk M, McQueen E, Mackay I, et al. Human Metapneumovirus in Lung Transplant Recipients and Comparison to Respiratory Syncytial Virus. Am J Respir Crit Care Med. 15 oct 2008;178(8):876‑81.

28. Hoppe BPC, de Jongh E, Griffioen-Keijzer A, Zijlstra-Baalbergen JM, IJzerman EPF, Baboe F. Human metapneumovirus in haematopoietic stem cell transplantation recipients: a case series and review of the diagnostic and therapeutic approach. The Netherlands Journal of Medicine. 2016;74(8):6.

29. Huijts SM, Coenjaerts FEJ, Bolkenbaas M, Werkhoven CH van, Grobbee DE, Bonten MJM. The impact of 13-valent pneumococcal conjugate vaccination on virus-associated community-acquired pneumonia in elderly: Exploratory analysis of the CAPiTA trial. Clinical Microbiology and Infection. 1 juill 2018;24(7):764‑70.

30. Hwang H, Kim Y, Park J-W, Jeong SH, Kyung SY. A Retrospective Study Investigating Risks of Acute Respiratory Distress Syndrome and Mortality Following Human Metapneumovirus Infection in Hospitalized Adults. Acute Crit Care. 31 mai 2017;32(2):182‑9.

31. Ibrahim S, Scott M, Bixler D, Pedersen R, Tripp J, Carter K, et al. Outbreaks of Human Metapneumovirus in Two Skilled Nursing Facilities — West Virginia and Idaho, 2011–2012. MMWR Morb Mortal Wkly Rep. 22 nov 2013;62(46):909‑13.

32. Johnstone J, Majumdar SR, Fox JD, Marrie TJ. Human Metapneumovirus Pneumonia in Adults: Results of a Prospective Study. Clin Infect Dis. 15 févr 2008;46(4):571‑4.

33. Kamboj M, Gerbin M, Huang C-K, Brennan C, Stiles J, Balashov S, et al. Clinical characterization of human metapneumovirus infection among patients with cancer. Journal of Infection. 1 déc 2008;57(6):464‑71.

34. Klein MB, Yang H, DelBalso L, Carbonneau J, Frost E, Boivin G. Viral pathogens including human metapneumovirus are the primary cause of febrile respiratory illness in HIV-infected adults receiving antiretroviral therapy. J Infect Dis. 15 janv 2010;201(2):297‑301.

35. Koo HJ, Lee HN, Choi SH, Sung H, Oh SY, Shin SY, et al. Human Metapneumovirus Infection: Pneumonia Risk Factors in Patients With Solid Organ Transplantation and Computed Tomography Findings. Transplantation. avr 2018;102(4):699‑706.

36. Koo HJ, Lee HN, Choi SH, Sung H, Kim HJ, Do K-H. Clinical and Radiologic Characteristics of Human Metapneumovirus Infections in Adults, South Korea. Emerg Infect Dis. janv 2019;25(1):15‑24.

37. Kwon et al. Microbiology from bronchoscopy in haematologic patients: comparison of stem cell recipients with nonrecipients [Internet]. Aspergillus and Aspergillosis. 2012 [cité 12 nov 2020]. Disponible sur: https://www.aspergillus.org.uk/conference_abstracts/microbiology-from-bronchoscopy-in-haematologicpatients-comparison-of-stem-cell-recipients-with-nonrecipients/

38. Li IWS, To KKW, Tang BSF, Chan K-H, Hui C-K, Cheng VCC, et al. Human metapneumovirus infection in an immunocompetent adult presenting as mononucleosis-like illness. Journal of Infection. 1 mai 2008;56(5):389‑92.

39. Li J, Wang Z, Gonzalez R, Xiao Y, Zhou H, Zhang J, et al. Prevalence of human metapneumovirus in adults with acute respiratory tract infection in Beijing, China. Journal of Infection. janv 2012;64(1):96‑103.

40. McCracken JP, Arvelo W, Ortíz J, Reyes L, Gray J, Estevez A, et al. Comparative epidemiology of human metapneumovirus- and respiratory syncytial virus-associated hospitalizations in Guatemala. Influenza and Other Respiratory Viruses. 2014;8(4):414‑21.

41. McManus TE, Marley A-M, Baxter N, Christie SN, O’Neill HJ, Elborn JS, et al. Respiratory viral infection in exacerbations of COPD. Respiratory Medicine. 1 nov 2008;102(11):1575‑80.

42. Mikulska M, Del Bono V, Gandolfo N, Dini S, Dominietto A, Di Grazia C, et al. Epidemiology of viral respiratory tract infections in an outpatient haematology facility. Ann Hematol. 1 avr 2014;93(4):669‑76.

43. Müller A, Klinkenberg D, Vehreschild J, Cornely O, Tillmann RL, Franzen C, et al. Low prevalence of human metapneumovirus and human bocavirus in adult immunocompromised high risk patients suspected to suffer from Pneumocystis pneumonia. Journal of Infection. mars 2009;58(3):227‑31.

44. Murali S, Langston AA, Nolte FS, Banks G, Martin R, Caliendo AM. Detection of respiratory viruses with a multiplex polymerase chain reaction assay (MultiCode-PLx Respiratory Virus Panel) in patients with hematologic malignancies. Leukemia & Lymphoma. 1 janv 2009;50(4):619‑24.

45. Niggli F, Huber LC, Benden C, Schuurmans MM. Human metapneumovirus in lung transplant recipients: characteristics and outcomes. Infectious Diseases. déc 2016;48(11‑12):852‑6.

46. Noel N, Rammaert B, Zuber J, Sayre N, Mamzer-Bruneel MF, Leruez-Ville M, et al. Lower Respiratory Tract Infection in a Renal Transplant Recipient: Do not Forget Metapneumovirus [Internet]. Case Reports in Transplantation. 2012 [cité 5 août 2019]. Disponible sur: https://www.hindawi.com/journals/crit/2012/353871/abs/

47. Oliveira RR, Machado AF, Tateno AF, Boas LV, Pannuti CS, Machado CM. Frequency of human metapneumovirus infection in hematopoietic SCT recipients during 3 consecutive years. Bone Marrow Transplantation. août 2008;42(4):265‑9.

48. Park S-Y, Baek S, Lee S-O, Choi S-H, Kim YS, Woo JH, et al. Efficacy of Oral Ribavirin in Hematologic Disease Patients with Paramyxovirus Infection: Analytic Strategy Using Propensity Scores. Antimicrob Agents Chemother. févr 2013;57(2):983‑9.

49. Peyrani P, Nahas A, Vanina Giovini, Meza Ortiz C, Wiemken T, Arnold FW, et al. Respiratory Viruses Are Significant Etiologic Agents In Hospitalized Patients With Lower Respiratory Tract Infections: Results From The Rapid Empiric Treatment With Oseltamivir Study (RETOS). In: A53 EPIDEMIOLOGY, RISK FACTORS, AND OUTCOMES OF RESPIRATORY INFECTIONS [Internet]. American Thoracic Society; 2012 [cité 2 août 2019]. p. A1796‑A1796. (American Thoracic Society International Conference Abstracts). Disponible sur: https://www.atsjournals.org/doi/abs/10.1164/ajrccm-conference.2012.185.1_MeetingAbstracts.A1796

50. Renaud C, Xie H, Seo S, Kuypers J, Cent A, Corey L, et al. Mortality Rates of Human Metapneumovirus and Respiratory Syncytial Virus Lower Respiratory Tract Infections in Hematopoietic Cell Transplant Recipients. Biol Blood Marrow Transplant. août 2013;19(8):1220‑6.

51. Samuel S, Nanjappa S, Cooper CD, Greene JN. Human Metapneumovirus Infection in Immunocompromised Patients. Cancer Control. oct 2016;23(4):442‑5.

52. Seo S, Gooley TA, Kuypers JM, Stednick Z, Jerome KR, Englund JA, et al. Human Metapneumovirus Infections Following Hematopoietic Cell Transplantation: Factors Associated With Disease Progression. Clin Infect Dis. 15 juill 2016;63(2):178‑85.

53. Shahda S, Carlos WG, Kiel PJ, Khan BA, Hage CA. The human metapneumovirus: a case series and review of the literature. Transpl Infect Dis. juin 2011;13(3):324‑8.

54. Souza JS, Watanabe A, Carraro E, Granato C, Bellei N. Severe metapneumovirus infections among immunocompetent and immunocompromised patients admitted to hospital with respiratory infection. Journal of Medical Virology. 2013;85(3):530‑6.

55. Span LFR, Dijk NM van, Linssen CFM, Mook WNKA van. Human metapneumovirus in immunocompromised patients with hAematological malignancies: awareness with caution and respect! [Internet]. Bone Marrow Transplantation. 2009 [cité 5 août 2019]. Disponible sur: https://link.galegroup.com/apps/doc/A198170167/AONE?sid=lms

56. Syha R, Beck R, Hetzel J, Ketelsen D, Grosse U, Springer F, et al. Humane metapneumovirus (HMPV) associated pulmonary infections in immunocompromised adults—Initial CT findings, disease course and comparison to respiratory-syncytial-virus (RSV) induced pulmonary infections. European Journal of Radiology. 1 déc 2012;81(12):4173‑8.

57. Tu C-C, Chen L-K, Lee Y-S, Ko C-F, Chen C-M, Yang H-H, et al. An outbreak of human metapneumovirus infection in hospitalized psychiatric adult patients in Taiwan. Scandinavian Journal of Infectious Diseases. 1 janv 2009;41(5):363‑7.

58. van Dijk N, Linssen C, Bussink M. Critical illness after horizontal nosocomial transmission of human metapneumovirus in the haematology ward. 2012;16(1):4.

59. Vanspauwen MJ, van Mook WN, Bruggeman CA, Bergmans DCJJ, Linssen CFM. Human metapneumovirus in bronchoalveolar lavage fluid of critically ill patients with suspected pneumonia. Intensive Care Med. 1 avr 2012;38(4):728‑9.

60. Vidaur L, Totorika I, Montes M, Vicente D, Rello J, Cilla G. Human metapneumovirus as cause of severe community-acquired pneumonia in adults: insights from a ten-year molecular and epidemiological analysis. Annals of Intensive Care. 24 juill 2019;9(1):86.

61. Wansaula Z, Olsen SJ, Casal MG, Golenko C, Erhart LM, Kammerer P, et al. Surveillance for severe acute respiratory infections in Southern Arizona, 2010-2014. Influenza Other Respir Viruses. mai 2016;10(3):161‑9.

62. Watanabe ASA, Carraro E, Candeias JMG, Donalísio MR, Leal É, Granato CFH, et al. Viral etiology among the elderly presenting acute respiratory infection during the influenza season. Revista da Sociedade Brasileira de Medicina Tropical. févr 2011;44(1):18‑21.

63. Weinberg A, Lyu DM, Li S, Marquesen J, Zamora MR. Incidence and morbidity of human metapneumovirus and other community-acquired respiratory viruses in lung transplant recipients. Transplant Infectious Disease. 2010;12(4):330‑5.

64. Widmer K, Zhu Y, Williams JV, Griffin MR, Edwards KM, Talbot HK. Rates of Hospitalizations for Respiratory Syncytial Virus, Human Metapneumovirus, and Influenza Virus in Older Adults. J Infect Dis. 1 juill 2012;206(1):56‑62.

65. Widmer K, Griffin MR, Zhu Y, Williams JV, Talbot HK. Respiratory syncytial virus- and human metapneumovirus-associated emergency department and hospital burden in adults. Influenza Other Respir Viruses. mai 2014;8(3):347‑52.

66. Wolfromm A, Porcher R, Legoff J, Latour RP de, Xhaard A, Fontbrune FS de, et al. Viral Respiratory Infections Diagnosed by Multiplex PCR after Allogeneic Hematopoietic Stem Cell Transplantation: Long-Term Incidence and Outcome. Biology of Blood and Marrow Transplantation. 1 août 2014;20(8):1238‑41.

67. Wong CKK, Lai V, Wong YC. Comparison of initial high resolution computed tomography features in viral pneumonia between metapneumovirus infection and severe acute respiratory syndrome. European Journal of Radiology. 1 mai 2012;81(5):1083‑7.

68. Ye C, Zhu W, Yu J, Li Z, Fu Y, Lan Y, et al. Viral pathogens among elderly people with acute respiratory infections in Shanghai, China: Preliminary results from a laboratory-based surveillance, 2012-2015. Journal of Medical Virology. 2017;89(10):1700‑6.

69. Yu X, Lu R, Wang Z, Zhu N, Wang W, Julian D, et al. Etiology and clinical characterization of respiratory virus infections in adult patients attending an emergency department in Beijing. PLoS ONE. 2012;7(2):e32174.
